# The Painless Treatment of the Drug Habit

**Published:** 1902-11

**Authors:** 


### TEXAS MEDICAL JOURNAL.

AUSTIN, TEXAS.

A MONTHLY JOURNAL OF MEDICINE AND SURGERY.

EDITED AND PUBLISHED BY
F. E. DANIEL, M. D.

ASSOCIATE EDITOR:
WITTEN BOOTH RUSS, M. D.,
San Antonio, Texas.

Published Monthly at Austin, Texas. Subscription price $1.00 a year in advance.

Eastern Representative: John Guy Monihan, St. Paul Building, 220 Broadway, New York City.

Official organ of the the West Texas Medical Association, the Houston District Medical Association, the Austin District Medical Society, the Brazos Valley Medical Association, the Galveston County Medical Society, and several others.

### THE PAINLESS TREATMENT OF THE DRUG HABIT.

**Dr. Lott's Method: Hyoscine Hydrobromate.**

In November last year we published a paper by Dr. M. K. Lott, of Cameron, Texas, under the title "The Drug Habit: Its Treatment." The paper had been presented before the Brazos Valley Medical Society, and was received with a rising vote of thanks and ordered published. It attracted wide attention and was extensively reproduced in our exchanges, and commented on editorially by many of them. We issued an extra large edition, but the demand for the paper soon exhausted it, and as we are still getting requests for it from all parts of the country or inquiries where it may be had, in response to this demand we have reproduced the paper in this issue.

Dr. Lott, at the request of its able editor, Professor Hobart Amory Hare, contributed a paper on the same subject to the *Therapeutic Gazette,* February, 1902. In the August 15, 1902, issue of that journal, Dr. R. E. Bering, of Tulane, California, gives the report of a case of alcoholism treated with hyoscine hydrobromate with success, and gives the minutest details of the treatment and the daily record of case, preceded by the following remarks:

"Having read with much interest the article in the February issue of the *Therapeutic Gazette,* by Dr. M. K. Lott, on the drink habit and its cure without pain, and knowing Dr. Lott by reputation, I decided to try his plan of treatment on my delirious patient. I must confess, however, that I started the treatment with considerable hesitancy. It is one thing for a man to break his own neck, but it is quite another to break it for him. This patient was delirious from whiskey, and I was preparing to start him on hyoscine and keep him intoxicated for forty-eight hours. However, I had faith, and started the treatment by giving ten grains of calomel Friday evening, March 15th, put the patient to bed, and administered the medicine as per the directions of Dr. Lott as follows: [Report omitted.—Ed.]

Dr. Hobart Amory Hare, the well-known author on therapeutics, also took up the treatment, and contributed to the June number (1902) of the *Medical News* (N. Y.) a valuable paper based on his experiences with the new treatment. I call attention to the conclusions arrived at by Dr. Hare: "First, the patients can take massive doses (of the hyoscine) for days at a time, as much as one-fourth grain each day hypodermatically, with no evil effect on any vital function; second, they suffer very slightly, if at all, from the immediate withdrawal of the morphine; and, third—and more surprising—the desire for the drug is largely, if not entirely, dissipated after a few days."

This is strong and valuable testimony.

In these days when the conscienceless quack is flooding the country with advertisements of secret remedies for the alleged painless cure of this most distressing habit, and fleecing the credulous victims, it is very gratifying to note the excellent work of this ethical physician who freely gives to the medical profession his treatment and every detail of his management of drug addictions, publishing it for the benefit of his colleagues. It is a matter of great pride to us, too, that that physician, an honored member of the State Medical Association and of several local societies, a gentleman who has contributed other valuable matter to the transactions of our societies, the discoverer of the most effective treatment for drug addictions, is a *native Texan.*

In the death of the lamented Adams the State loses one of its most valued citizens and the medical profession one of its most zealous and distinguished members. Dr. William Arnold Adams, of Fort Worth, died suddenly at his home in that city on the 15th of October (ult.). He was a native of Georgia and was in his forty-ninth year. Dr. Adams received his medical degree from the Medical Department of the University of Georgia in 1876, and the degrees A. M. and LL. D. from Mercer University, Georgia. At the time of his death Dr. Adams was Emeritus Professor of Medicine in the Medical Department of Fort Worth University, and filled many other positions of honor and usefulness. He was medical inspector for Texas, Oklahoma and Indian Territory for the Equitable Insurance Co., and was surgeon of several railroads. Dr. Adams had been Vice-President of the Texas State Medical Association. His early death is most sincerely mourned by thousands of attached friends.

**Dr. William Arnold Adams.**

---

Dr. W. M. Vertrees, of Nashville, died recently at his home from "nervous exhaustion." Dr. Vertrees was prominent in the medical profession, and was widely known. In 1874 he was associated with Dr. Paul F. Eve, Sr., and was one of the founders of the present flourishing and popular Medical Department of the University of Tennessee. In this school he held the Chair of Materia Medica continuously for twenty-five years. Aged 76 years.

**Dr. W. M. Vertrees.**

---

The volume of Transactions of the State Medical Association just turned out by the publishers, the great printing establishment of Von Boeckmann, Schutze & Co., Austin, certainly excels any of the preceding volumes, and, in fact, anything of the kind we have seen from anywhere or any society. It is a credit to the Association, a cerdit to its zealous, capable and popular Secretary, Dr. H. A. West, of Galveston, a credit to the Von Boeckmann's, to Austin, and in fact, to Texas. If any one should be so benighted as to not know that in Texas we are up to date in printing and binding—as well as in medicine and medical societies, medical politics and in sanitation—let him view this handsome volume, and read the proceedings of the great T. S. M. A. and the papers. The volume contains nearly 600 pages. It is printed on a superior quality of paper, in clear type, and is beautifully bound in maroon cloth and gilt. It is

**Transactions of The State Medical Association of Texas.**

a model of typography and the book binders' art. There are more than a score of illustrations accompanying the text of the papers.

Where so many papers are excellent, able, interesting, valuable and instructive, it would be unfair—indeed, it would be impossible —to select any one or more for especial commendation. I will say, however, that the paper on "The Venomous Reptiles of Texas," by Dr. Crouse, of Victoria, copiously illustrated, is of exceptional value and will become a standard, as I know of no work of the kind near so complete and reliable. The paper of Dr. J. W. McLaughlin, Professor of Medicine in the Medical Department of the University of Texas, is one of great practical value. It treats of the importance of and the means to be employed in the prevention of tuberculosis in private practice. That it the title of the paper. Every physician should read it. Unfortunately many practitioners are not sufficiently impressed with the fact that consumption is *not* a *contagious* disease (that is, a disease than can be transmitted by contact), but is a dangerous and easily communicable and, therefore, an *infectious* disease; one, moreover, that is easily preventable; that the danger of auto-reinfection, as well as infection of well persons, lies in the *sputa* and other secretions or excretions, and that if these be promptly destroyed the disease is robbed of its dangers. Let this valuable truth be industriously disseminated, and the first step will have been taken to diminish the dangers, arrest the spread, and allay the almost panic that is caused by the general prevalence of this most dreaded and deadly disease.

---

**The Public and The Doctor: Dr. Hadra's book.**

We have received from the author a copy of a small cloth bound book under the above title, and have read it with interest and profit. It will be found to be a great aid to the practicing physician and a valuable factor in the "campaign of education" now in progress; for it is not all of a doctor's mission and duty to give drugs; he must enlighten his patrons on a thousand things that nearly concern their health, and especially on sanitation and hygiene. He cannot deliver a lecture to every family. Indeed, they would forget most of it if he did, before he had reached his buggy; but he can leave with each family a copy of this little book, and they will study it and profit by it. The people know nothing, absolutely nothing, of medical matters, nor of hygiene, nor of sanitation, and hence they are "easy marks" for the quack, who, with the aid of the villainous advertisements in nearly every newspaper, proceeds to rob, and slowly murder, scores of the ignorant and credulous. The little book is intended to enlighten the families in whose hands it is placed by their physician on these subjects; to open their eyes to the quack and his methods and nostrums; and to aid the physician in preaching the gospel of cleanliness, personal and household hygiene, and in fact to help him in his many difficulties with the ignorant and uninformed. Dr. Hadra's book is strictly ethical, and I commend it to my readers with confidence. Its author—a man of international reputation as a surgeon, a teacher, an author; an ex-President of the Texas State Medical Association; a time-honored worker in the cause of legitimate medicine—is too well known to the readers of the JOURNAL to need any words of approval or endorsement at my hands. Well known throughout the South, he is especially esteemed and appreciated in Texas, where those who know him best love and admire him most.

This little book is sold at only 50 cents. Send your order for one or a dozen copies to the author—who is also the publisher—Dr. B. E. Hadra, Dallas, Texas, or to this office.

---

**The State Board Medical Examiners.**

The State Board of Medical Examiners for Texas met at Dallas on the 7th of Ooctober, ult., and examined twenty-six applicants for license to practice. Of this number eight failed to pass a satisfactory examination—a pretty heavy percentage of rejections, nearly one-third. The Board will not meet again until April 20, 1903. The meeting will be held at Austin, and will be the last meeting of the Board as now constituted.

---

**The West Texas Medical Society.**

The famous West Texas Medical Society has certainly shaken up the dry bones in this section and infused a newness of life into ye doctors. Great Scott, but that was a splendid meeting—see Russ's report—and a banquet to make a fellow's head swim! The good things they gave us to eat and drink were enough to make a fellow forget his sins a whole year. It was a feast of wit and a flow of wine; a feast of mirth and merriment and a flow of Mumm (mum's the word). It is good for a pious old doctor to get away from home once in a while; to forget his interesting cases; to quit looking solemn; to cut out Johny and his mumps and Sissy and her measles, to tell the grizzley old bores with their rheumatism to go to grass, and go down to San Antonio and take a time with the "boys." It makes one feel better; it takes the wrinkles out of his face and the aches out of his j'ints. A doctor is something like his horse, after all—begging the horse's pardon. He gets burnt out on dry feed and needs a little "roughness"—long forage; and then, too, to have the harness taken off and run in the pasture and—well—you all know how it is yourselves. Other societies must follow the example set by the West Texas and stir the doctors up and make them turn out —turn out.

---

DR. ROBERT T. MORRIS, of Houston, was married October 16th (ult.) at Chillicothe, Mo., to Miss Bertha M. Reynolds of that city. Dr. Morris was born and raised in Austin, and he is a credit to his "bornin."

---

DR. J. D. OSBORN, JR., of Oklahoma City, was married in Cleburne, Texas, October 8th (ult.), to Miss Brown, of Cleburne. At the same time and place Miss Irene Osborn was married to Mr. Blair, both of Cleburne. Dr. Osborn and Mrs. Blair are son and daughter of our handsome and distinguished friend Dr. J. D. Osborn, ex-President of the Texas State Medical Association, a genial and delightful fellow.

